# Imaging analysis reveals budding of filamentous human metapneumovirus virions and direct transfer of inclusion bodies through intercellular extensions

**DOI:** 10.1128/mbio.01589-23

**Published:** 2023-09-08

**Authors:** Farah El Najjar, Santiago Restrepo Castillo, Carole L. Moncman, Cheng-Yu Wu, Eduardo Isla, A. Catalina Velez Ortega, Gregory I. Frolenkov, Nicolas Cifuentes-Munoz, Rebecca Ellis Dutch

**Affiliations:** 1 Department of Molecular and Cellular Biochemistry, University of Kentucky College of Medicine, Lexington, Kentucky, USA; 2 Instituto de Ciencias Biomédicas, Facultad de Ciencias de la Salud, Universidad Autónoma de Chile, Santiago, Chile; 3 Department of Physiology, University of Kentucky College of Medicine, Lexington, Kentucky, USA; St. Jude Children's Research Hospital, Memphis, Tennessee, USA

**Keywords:** pneumovirus, HMPV, viral spread, respiratory viruses

## Abstract

Human metapneumovirus (HMPV) can spread between cells through budding of virus particles or direct cell-to-cell spread. A network of budding filaments and intercellular extensions forms in HMPV-infected cells; however, the involvement of these structures in direct cell-to-cell spread of infection remains to be investigated. Utilizing advanced imaging techniques, we show that budding filaments contain the viral RNA genome, and not the antigenome, and become enriched with HMPV F protein as infection progresses. Multiple filaments containing the F protein on their surface were seen emanating from one cell to contact a neighboring cell, strongly suggesting that these are budding filamentous HMPV virions. In addition, changes in the morphology and properties of intercellular extensions following HMPV infection were detected. We show that intercellular extensions allow the transfer of a cytosolic dye only in HMPV-infected cells. In addition, using live imaging, we provide novel evidence of the direct passage of inclusion bodies from cell-to-cell across intercellular extensions. These results provide novel insights into the direct cell-to-cell spread of HMPV and suggest that this mode of transmission can occur through different mechanisms.

**IMPORTANCE:**

Human metapneumovirus is an important respiratory pathogen that causes significant morbidity and mortality, particularly in the very young, the elderly, and the immunosuppressed. However, the molecular details of how this virus spreads to new target cells are unclear. This work provides important new information on the formation of filamentous structures that are consistent with virus particles and adds critical new insight into the structure of extensions between cells that form during infection. In addition, it demonstrates for the first time the movement of viral replication centers through these intercellular extensions, representing a new mode of direct cell-to-cell spread that may be applicable to other viral systems.

## INTRODUCTION

Viruses are obligate intracellular parasites that utilize cellular resources to generate virions. To spread infection, cell-free virions travel across extracellular environments to reach uninfected cells and initiate a *de novo* infection. There is strong evidence demonstrating that viruses can also spread directly from cell-to-cell (1). Direct cell-to-cell spread generally involves a dramatic remodeling of the cellular architecture, which is induced by viral infection, resulting in the formation of syncytia, intercellular extensions, or intercellular pores (2). Moreover, some mechanisms of cell-to-cell spread allow viruses to disseminate in the presence of neutralizing antibodies or extracellular immune system components (3, 4). This intriguing observation suggests that direct cell-to-cell spread can occur directly between the cytoplasms of two cells without formation of the virus.

Human metapneumovirus (HMPV) is a respiratory pathogen responsible for up to 7% of respiratory infections in pre-pandemic times (5). As the COVID-19 pandemic progressed, infections by HMPV and other respiratory viruses dramatically decreased, likely due to the social distancing and masking policies reducing overall viral transmission. However, several reports have shown that HMPV continued to circulate at low levels, including in coinfections with SARS-CoV-2, which in some cases led to severe disease and death of pediatric patients (6 – 8). Data obtained from the national respiratory and enteric virus surveillance system show that HMPV circulation started to rise in 2022 up to levels found in pre-pandemic times. To date, no licensed vaccines or antivirals are available to prevent infections caused by HMPV.

HMPV is a member of the *Pneumoviridae* family, characterized by the presence of a negative-sense single-stranded RNA genome (9). After virus entry, the viral ribonucleocapsid is transported to the vicinity of the nuclei to start replication and transcription (10). Both processes are performed by the RNA-dependent RNA polymerase complex, composed of the viral RNA (vRNA), nucleoprotein (N), phosphoprotein (P), polymerase (L), and accessory proteins (11 – 13). Similar to other members of the *Mononegavirales* order including measles virus, Ebola virus, and rabies virus, infection by pneumoviruses results in the formation of cytosolic inclusion bodies (IBs), organelles with liquid-like properties where viral genome replication and transcription takes place (10, 14 – 21). Within IBs, the pneumoviral RNA polymerase, together with the polymerase cofactors, uses the vRNA as a template for synthesis of full-length positive-sense antigenome and viral messenger RNAs (11, 13, 22, 23). Antigenome RNA is then used as a template by the RNA polymerase for synthesis of vRNA. Budding of HMPV particles from the plasma membrane occurs once all viral components concentrate at assembly sites (12). However, release of HMPV particles from the apical side of three-dimensional airway tissues has been shown to be significantly less efficient than release of human respiratory syncytial virus (HRSV) particles (3). Infected HMPV cells can fuse to neighboring cells by the action of the F protein, giving rise to large multinucleated cells named syncytia (24 – 26). Syncytia formation has been proposed as a mechanism of direct cell-to-cell spread that could function alongside with release of individual virions (1). However syncytia formation was not observed in HMPV-infected three-dimensional airway tissues (3). It has also been shown that syncytia formation is low in differentiated primary bronchial cell cultures infected with HRSV and absent in animals (27). Interestingly, long intercellular extensions are observed in HMPV-infected airway tissues, which were proposed to be associated with direct cell-to-cell spread (3). Formation of long intercellular extensions in HMPV-infected cells has additionally been demonstrated *in vitro* (4). Besides formation of intercellular extensions, HMPV-infected cells display abundant shorter actin-based branching filaments, which may represent budding viruses. Remarkably, up to 50% of viral spread can occur in the presence of HMPV-neutralizing antibodies and in the absence of appropriate attachment factors (4), indicating that cell-to-cell spread is important for HMPV infection processes. Filopodia-like intercellular structures have been shown to facilitate viral spread of RSV in lung epithelial cells (28). In addition, other viruses including paramyxoviruses, influenza, SARS-CoV-2, and retroviruses can also use filaments and intercellular extensions for direct cell-to-cell spread (1, 4, 28 – 32).

Cell-to-cell spread has been studied in many viral systems, yet the exact mechanisms of infection dissemination are not completely clear. Here, a detailed analysis combining electron and confocal microscopy permitted us to characterize both branching filaments and intercellular extensions that protrude from HMPV-infected cells. Live cell imaging demonstrated that extensions from HMPV-infected cells can open toward neighboring cells, allowing spread of a cytosolic dye. Furthermore, pores opened between cells can support direct transfer of HMPV ribonucleocapsids and large IBs between cells. Direct cytosolic transfer of IBs between cells is a mechanism that, to our knowledge, has not been reported before. The mechanism by which HMPV can disseminate genetic material between cells represents a novel route of infection.

## MATERIALS AND METHODS

### Cell lines

BEAS-2B cells, obtained from ATCC, were grown in bronchial epithelial cell growth basal medium (BEBM) supplemented with the BEGM SingleQuots supplement and growth factors kits (Lonza) as per ATCC instructions. For live cell imaging, BEAS-2B cells were cultured on noncoated glass-bottomed petri dishes in Opti-MEM supplemented with 2% (vol/vol) fetal bovine serum (FBS). Vero cells were grown in Dulbecco’s modified Eagle’s medium (DMEM) containing 10% FBS (vol/vol).

### Virus propagation and infection

Wild-type (WT) HMPV and both recombinant, enhanced green fluorescent protein (EGFP)-expressing HMPV (rgHMPV) strain CAN97-83 (genotype group A2) and mCherryP-expressing HMPV (strain JPS02-76) were propagated and titered on Vero cells as previously described (18). For infection, cells were washed 3× with phosphate buffered saline (PBS) and then inoculated with HMPV at the desired multiplicity of infection (MOI) in Opti-MEM medium. Cells were incubated for 3 h, washed once with PBS, and fresh media was then added.

### Scanning electron microscopy

Cells undergoing conventional scanning electron microscopy (SEM) imaging were fixed in 3% glutaraldehyde in 0.1 M cacodylate buffer (Electron Microscopy Sciences). Cells undergoing immunogold SEM imaging were fixed in 4% paraformaldehyde and 0.2% glutaraldehyde followed by blocking in 1% (vol/vol) normal goat serum (NGS) and incubation with 54G10 anti-HMPV F antibody, kindly provided by Dr. John Williams, University of Pittsburgh, at 4°C overnight. Colloidal gold-affinipure goat anti-human IgG (12 nm) was used for gold immunolabeling and cells were post-fixed in 2.5% glutaraldehyde in 0.1 M cacodylate buffer. Fixed samples were dehydrated with graded series of ethanol, critical point dried from liquid CO_2_ and sputter coated with 5 nm platinum (conventional SEM) or 2 nm palladium (immuno-SEM). Imaging was performed using a ZEISS EVO MA 10 microscope (conventional SEM) or a FEI Helios Nanolab 660 (immuno-SEM).

### Dye transfer

Glass micropipettes were pulled from borosilicate glass capillaries (World Precision Instruments) using the P-1000 heating filament puller (Sutter Instrument). Typically, pipettes exhibiting a resistance of 10 to 12 MΩ in the bath were used. The intracellular solution used in the pipettes contained (in mM): KCl (12.6), KGlu (131.4), MgCl_2_ (2), EGTA (0.5), K_2_HPO_4_ (8), KH_2_PO_4_ (2), Mg_2_-ATP (2), and Na_4_-GTP (0.2) supplemented with 250 µM of the fluorescent dye Alexa Fluor 594 (Molecular Probes). The osmolarity and pH of the intracellular solution was adjusted to match that of the bath solution (typically, 310 mmol/kg and pH 7.4). Whole-cell patch configuration was achieved to allow for the diffusion of the dye into the cytoplasm of a particular cell. Dye “injection” and imaging were performed at room temperature using an upright Olympus BX51WI spinning disc confocal microscope equipped with a 40 × 0.8 NA objective (Olympus), a nanopositioning system for focal plane control (Mad City Labs), an Evolve 512 EMCCD camera, a U-RFL-T mercury arc lamp (Olympus), and an MP-285 micromanipulator (Sutter Instrument). Only the cells that were shown to make physical contact with the injected cell in bright field examination were used for quantification purposes.

### Live cell imaging

VeroE6 cells were seeded in six well glass-bottom culture plates and infected with HMPV-mCherryP (MOI 3) on the following day. Cells were kept at 37°C in a 5% CO_2_ atmosphere until imaging. Images were acquired in a LionHeartFX fluorescence microscope using a 60× oil immersion objective. At 72 hpi infected cultures were imaged for 24 h, with images taken every 1 min. At least five different infected cells were imaged per condition. For live imaging of BEAS-2B cells, cells were grown in 35 mm glass-bottom dishes and the following day inoculated with rgHMPV (MOI 10). At 3 hpi, cells were transfected with a pCAGGS plasmid containing the HMPV mCherry-P gene using Lipofectamine 3000 (ThermoFisher, L3000008) according to the manufacturer’s protocol. Media was changed the following day and at 48 hpi, live imaging was performed using a Nikon A1 confocal microscope. A 40× oil immersion objective was used and sequential images were acquired every 1 min. Images were collected and video was created by NIS Elements software.

### Quantitative analysis of extensions and filaments

The length and width of intercellular extensions and filaments were measured manually using the line measurement tool in NIH ImageJ analysis tool. The width was determined in the middle of a filament or extension and the length was taken from the point the filament or extension protruded from the cell until the tip. For gold nanoparticle (GNP) quantification, images were taken for each of the specified cell structures and manually analyzed in ImageJ. Briefly, five squares (1 µm × 1 µm) were drawn for each image, the number of GNPs in each square counted and their average density (GNP/µm^2^) determined. Degree of filamentous branching was assessed using the Sholl analysis tool in ImageJ by drawing a series of concentric circles with 1 µm radii intervals (starting at 5 µm to exclude the cell body) and determining the number of intersections at each circle. An average of 3–5 cells/condition was analyzed for each of the aforementioned parameters and GraphPad Prism version 9.3.1 (GraphPad Software, San Diego, California, USA; www.graphpad.com) was used to perform statistical analysis.

### Quantitative analysis of fluorescence *in situ* hybridization experiment

BEAS-2B cells inoculated with rgHMPV (MOI 3) were fixed at 24 hpi with 4% paraformaldehyde and processed for fluorescence *in situ* hybridization (FISH) staining as previously described (10). Fifty image stacks were collected of HMPV-infected BEAS-2B cells probed with vRNA and +RNA FISH probes. After correction of the far-red signal (+RNA) for optical aberrations, a maximum intensity profile was generated for each image stack and thresholds were generated for each probe. Using the morphological criteria defined in El Najjar et al. (4), regions of interest (ROIs) were outlined and divided into the categories of extensions or filaments. NIS Elements was used to generate the sum intensity for each signal within the ROIs. To standardize the measurements, the sum intensity of the +RNA was divided by the sum intensity of the vRNA and GraphPad Prism version 9.0.2 (GraphPad Software, San Diego, California, USA; www.graphpad.com) was used to perform statistical analysis.

## RESULTS

### Intercellular extensions in HMPV-infected cells are covered with filaments and vesicular bodies

**Fig 1:**
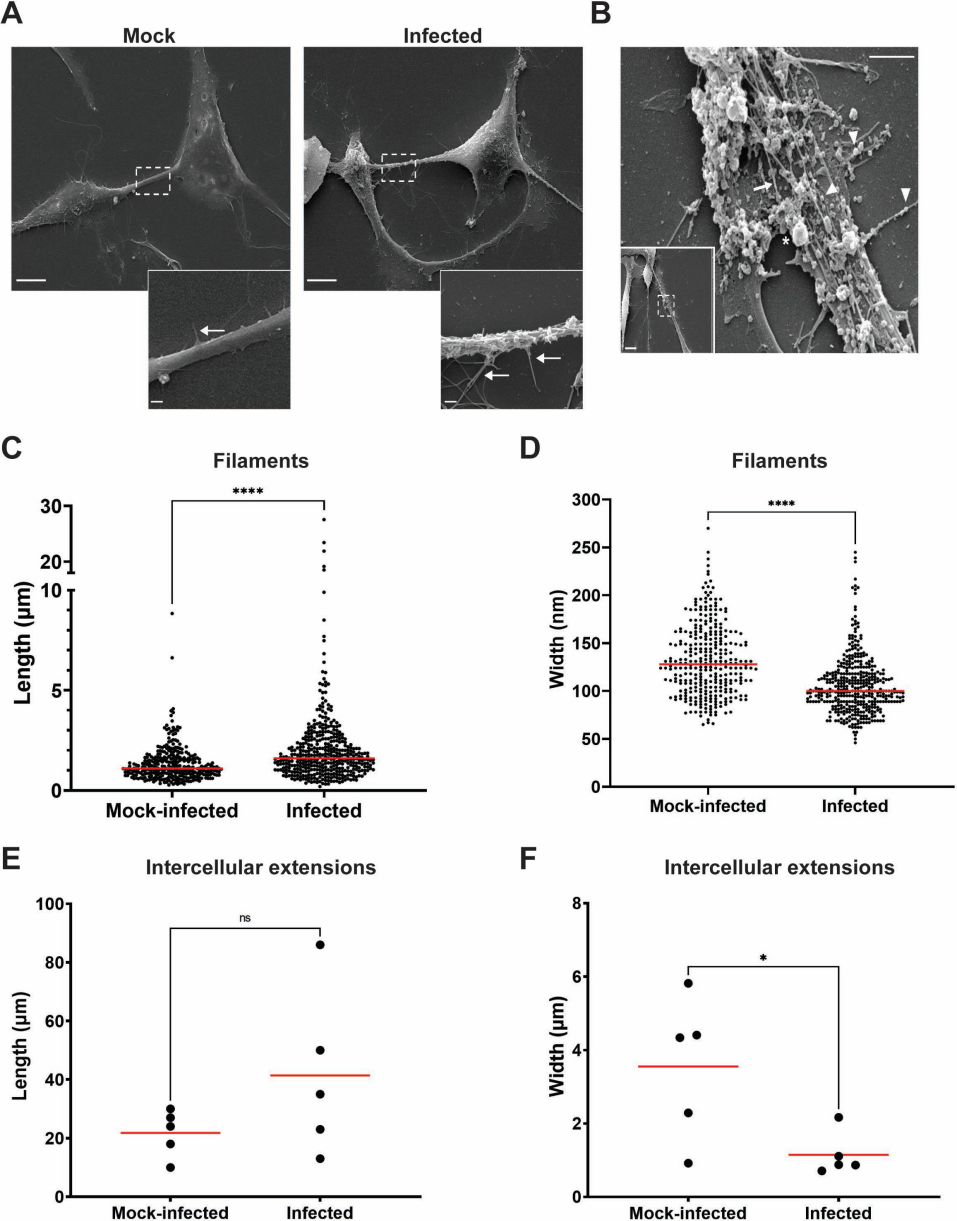
Intercellular extensions in HMPV- and mock-infected cells differ in morphology and size. BEAS-2B cells were mock infected or infected with HMPV at an MOI of 3 and 24 hpi cells were fixed and prepared for electron microscopy. (**A**) Scanning electron microscopy images showing intercellular extensions forming between two cells in mock- and HMPV- infected cells. Inset shows magnification of the boxed areas. Arrows indicate filaments coming off an intercellular extension in mock and infected cells. Scale bar = 10 µm. Scale bar in insets = 1 µm. (**B**) Intercellular extensions in infected cells are covered with filaments (arrow), and with small (arrowhead) and large (asterisk) vesicles. Scale bar = 2 µm. Scale bar in inset = 10 µm. (**C and D**) Quantification of length and width of filaments in mock- and HMPV- infected cells (320 filaments/mock-infected cells and 413 filaments/infected cells). (**E and F**) Quantification of length and width of intercellular extensions in mock- and HMPV- infected cells (*n* = 5 intercellular extensions /mock-infected cells and 5/intercellular extensions/infected cells). Statistical analysis was performed using unpaired *t*-test (****P < 0.0001, *P < 0.05, ns = not significant).

HMPV infection in the bronchial epithelial human cell line BEAS-2B results in the formation of filopodia-like intercellular extensions and a network of cell-associated filaments (4). To characterize the nature of these structures in high resolution, we performed SEM imaging on mock- and HMPV-infected BEAS-2B cells. Filaments and intercellular extensions were differentiated based on their diameter as previously described (4). Intercellular extensions emanating from one cell and connecting to a neighboring cell were seen in both mock-infected and infected cells as previously shown (4), but significant differences were observed. Extensions in mock-infected cells had a smooth surface with few small filaments protruding from the extensions (Fig. 1A). In infected cells however, the surface of intercellular extensions was covered with filaments that were arranged in a parallel fashion from which smaller filaments extended (Fig. 1A and B). In addition, pleomorphic vesicles of different sizes were seen along the length of the extensions only in infected cells. Small vesicles were also detected on filaments coming off the extensions (Fig. 1B). The nature and properties of these vesicles remain to be explored. We then assessed how HMPV infection affected the size of the extensions and filaments by quantifying the length and width of these structures in HMPV- and mock-infected cells. Quantification of peripheral filaments showed thinner and longer filaments in HMPV-infected cells, with an average of 106 nm and 2.2 µm, respectively, compared to 130 nm and 1.3 µm in noninfected cells (Fig. 1C and D). Intercellular extensions in infected cells were significantly thinner (Fig. 1F) and longer, almost double the length of extensions in mock-infected cells with an average of around 40 µm (Fig. 1E). It is possible that some of the intercellular extensions can be the result of completion of the process of cell division; however, in HMPV-infected cells the extensions are longer with more branching indicating exploitation by the virus. These results show that while intercellular extensions and filaments are present in uninfected BEAS-2B cells, HMPV infection results in changes in the ultrastructure and size of these structures.

### Budding filaments that are enriched in F protein and vRNA touch neighboring cells

**Fig 2:**
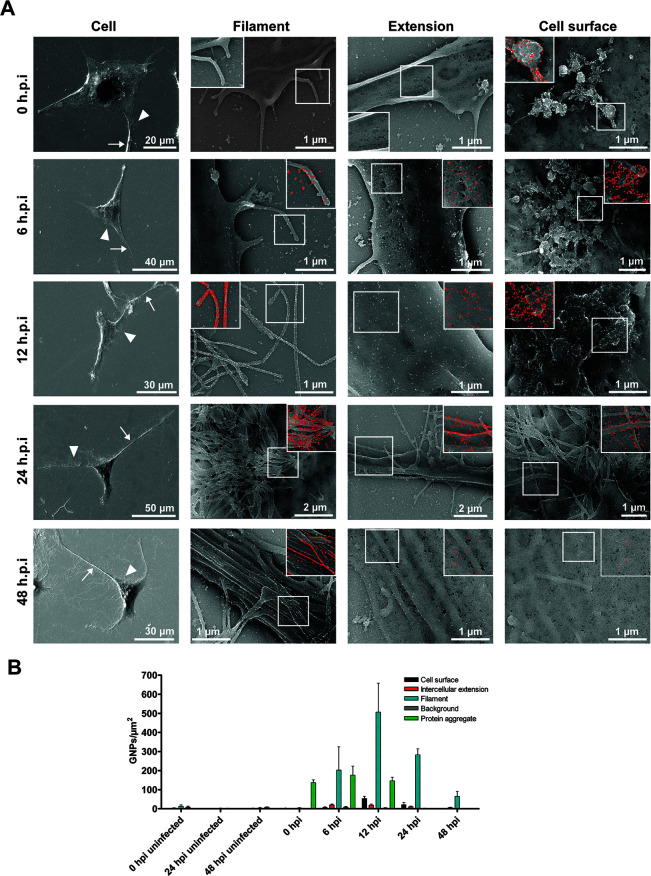
HMPV F protein appears in budding filaments as infection progresses. BEAS-2B cells were inoculated with HMPV at an MOI of 4 and 4 h later cells were either fixed (0 hpi) or incubated for the indicated time points prior to fixing. Cells were then processed for immunogold labeling using an antibody for HMPV F protein. (**A**) SEM images show cellular distribution of F protein in filaments, intercellular extensions and at the cell surface. Insets show immunogold particles false-colored with red. Arrows indicate intercellular extensions and arrowheads indicate filaments. (**B**) Quantification of gold nanoparticles on the indicated cell structure was performed manually using ImageJ. The graph shows mean ± SD for five cells at each time point.

**Fig 3:**
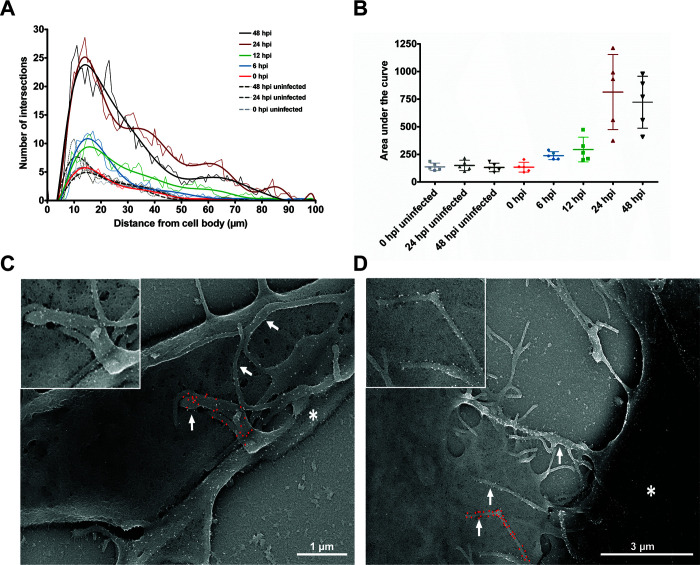
Filamentous branching increases significantly with time in HMPV-infected cells and filaments protrude from intercellular extensions and the cell body. (**A and B**) BEAS-2B cells were inoculated with HMPV at an MOI of 4 and incubated for the indicated time points prior to fixing. Cells were then prepared for SEM and images were acquired and processed. A total of five cells per time point were traced using the NeuronJ plugin of NIH ImageJ analysis tool and the degree of branching determined using Sholl analysis. (**B**) Graph shows mean ± SD for five cells at each time point. (**C and D**) SEM images of F protein immunogold-labeled cells showing point of contact of filaments protruding from an intercellular extension (**C**) or cell body (**D**) of an infected cell and touching an uninfected cell. White arrows indicate filaments containing F protein and asterisk indicates an infected cell. Immunogold particles were false-colored with red in one of the filaments from each infected cell. Insets show magnified filaments with the native white immunogold labeling.

**Fig 4:**
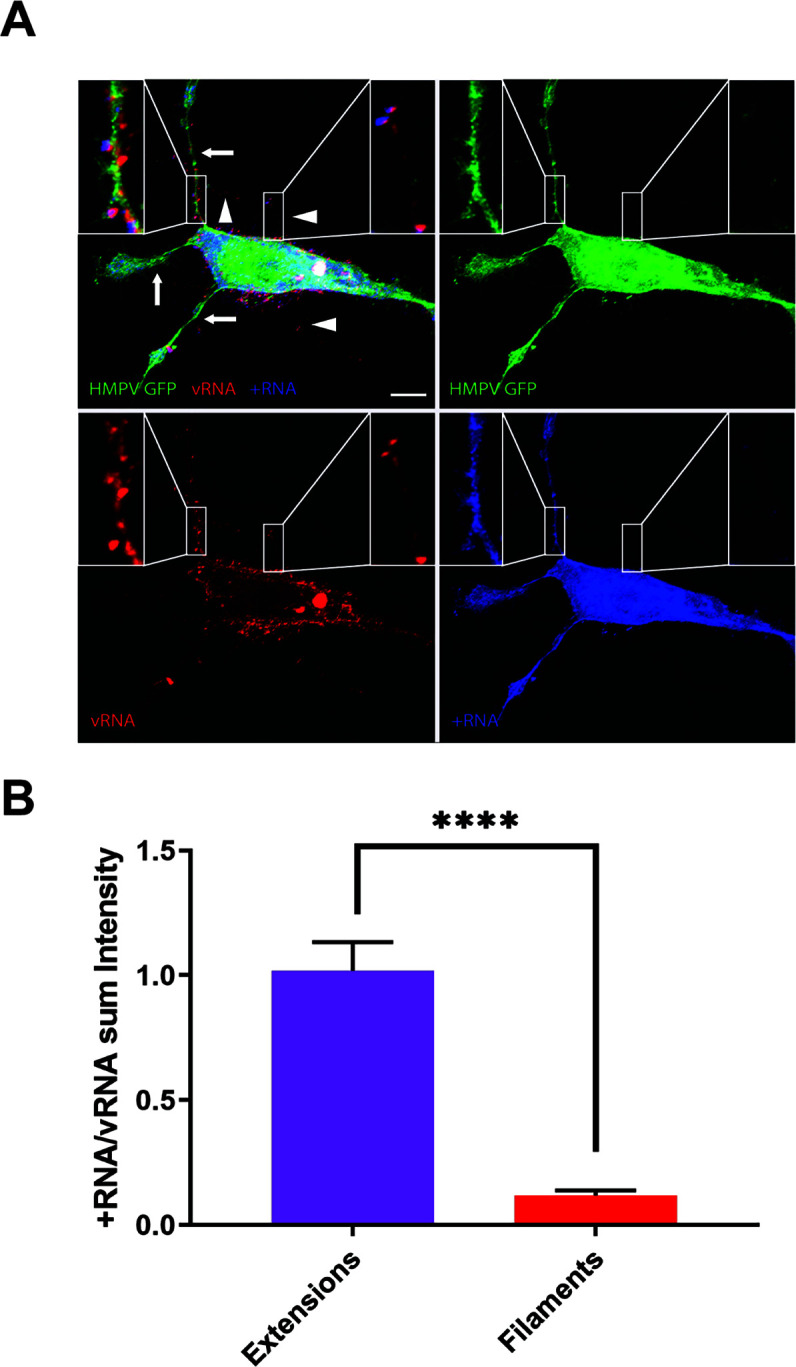
vRNA is concentrated in budding filaments and not in extensions. (**A**) BEAS-2B cells were inoculated with rgHMPV at an MOI of 3. Cells were fixed at 24 hpi and FISH was performed to detect vRNA (red) and +RNA (blue). Insets show localization of GFP, vRNA, and +RNA in extensions (left inset) and filaments (right inset). (**B**) Quantification of the sum intensity for each signal in extensions and filaments was performed using NIS Elements software and statistical analysis was done using unpaired *t*-test (****P < 0.0001). A total of 66 extensions and 89 filaments were used for this analysis.

Several respiratory viruses, including influenza virus, parainfluenza, and RSV (25 – 29), bud in a filamentous form, and HMPV forms filaments in LLC-MK2 cells (30). Branching HMPV filaments that bud off from the cell body and intercellular extensions in BEAS-2B cells contain the viral proteins N, P, F, and M, and Stochastic Optical Resolution Microscopy imaging revealed an organized localization of M and N within these filaments (4). We evaluated the ultrastructure of the budding filaments at different times post-infection. To identify infected cells, we used immunogold HMPV F protein labeling. At 0 hpi, short protruding filaments were seen in cells, with HMPV F protein mainly localized to vesicles that could potentially correspond to aggregated virus particles (Fig. 2). As infection progressed, F protein was detected in intercellular extensions and the cell surface but was primarily concentrated in the branching filaments that protrude from the cell body and extensions (Fig. 2A and B). The filaments became longer and formed a complex branched network at 24 and 48 hpi as previously described (4). Quantifying the degree of branching of the filamentous network using Sholl analysis showed that branching increased at 24 and 48 hpi (Fig. 3). The expression of F protein on the filaments decreased as the degree of branching increased, likely corresponding to F protein being distributed on a larger membrane area rather than lower expression levels (Fig. 2B). Filaments covered with F protein mediated points of contact between infected cells and neighboring cells as seen in Fig. 3. These filaments came from infected cells, either from intercellular extensions or directly from the cell body, and contacted neighboring uninfected cells (Fig. 3C and D). To further characterize the budding filaments, and better understand how they relate to the intercellular extensions, we performed FISH staining on BEAS-2B cells 24 h after HMPV infection. Two different sets of probes were used specific to either positive-sense (+RNA) that corresponds to both antigenome and mRNA, or probes specific to vRNA as previously described (8). Both RNA subpopulations were localized in the cytoplasm and in the three intercellular extensions seen protruding from an infected cell. Interestingly, however, little +RNA staining was observed in the shorter filaments branching off from the cell body or from the intercellular extensions (Fig. 4A). Quantification of the intensity of the signal for vRNA and +RNA in intercellular extensions and filaments showed that the ratio of +RNA to vRNA is significantly higher in intercellular extensions compared to filaments, indicating that +RNA is mainly located in extensions and not in filaments (Fig. 4B). +RNA corresponds to antigenome (which acts as a template for the replication of vRNA) and viral mRNAs that are needed for translation of HMPV proteins (8), only -RNAs are packaged. Collectively these results show that budding filaments which protrude from the cell body or intercellular extensions contain HMPV F protein and vRNA, strongly suggesting that they correspond to filamentous HMPV virus particles budding from the cell surface.

### BEAS-2B cells infected with HMPV exhibit cytoplasmic communications with neighboring cells

**Fig 5:**
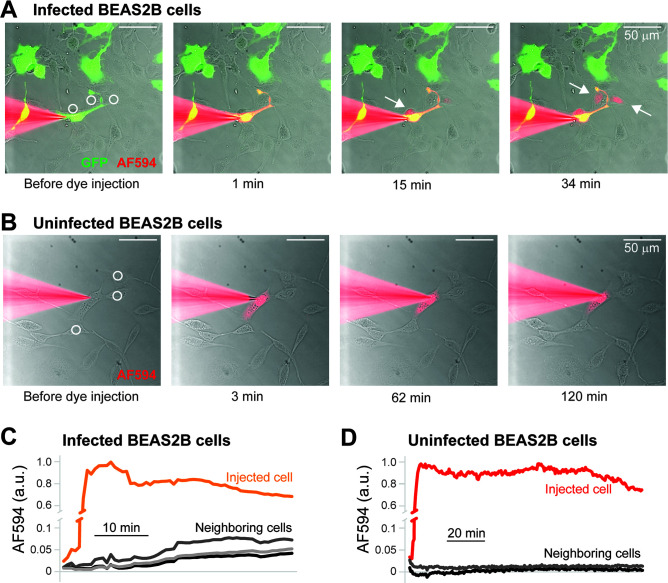
Evidence of cytoplasmic communications between HMPV-infected cells and neighboring cells. (**A**) Time-lapse imaging of BEAS-2B cells infected with rgHMPV. Panels immediately before and from several time points after the injection of a GFP+ cell (green) with the Alexa Fluor 594 dye (AF594, red) are shown. Arrows point to neighboring cells that showed transfer of AF594. (**B**) Time-lapse imaging of mock-infected BEAS-2B cells, showing the injection of AF594 into a cell but no evidence of dye transfer to any of its neighboring cells. (**C and D**) Quantification of AF594 fluorescence in the injected GFP+ infected BEAS-2B cell (**C**) and the injected mock-transfected cell (**D**) and in three neighboring cells each, highlighted by white circles in panels A and B.

Some intercellular extensions that form between cells are open-ended, allowing the passage of cytosolic contents between two cells, while others are close ended (29, 33). To test whether the intercellular extensions induced in HMPV-infected cells allow the transmission of materials through either temporary or continuous cytoplasmic communications, we microinjected HMPV-infected (GFP+) BEAS-2B cells with the fluorescent dye Alexa Fluor 594 and performed time-lapse imaging to assess any dye transfer to noninjected neighboring cells that were in physical contact (Fig. 5A and C). After nine independent dye injections of GFP +BEAS-2B cells, we observed the transfer of the Alexa Fluor 594 dye into 11 out of 44 cells that were making physical contact with the injected cells. The dye transferred to both GFP+ (infected, *n* = 2) and GFP− (noninfected, *n* = 9) cells, which were making physical contact with the injected cell. In contrast, after five independent dye injections of mock-infected BEAS-2B cells (Fig. 5B and D) only 1 out of 14 cells making physical contact with the injected cell showed Alexa Fluor 594 dye transfer (*P* = 0.0586). These results indicate that intercellular extensions formed in HMPV-infected cells can provide intercellular cytoplasmic communications with neighboring cells to potentially allow the transfer of HMPV infectivity.

### HMPV IBs pass directly from cell-to-cell through intercellular extensions

**Fig 6:**
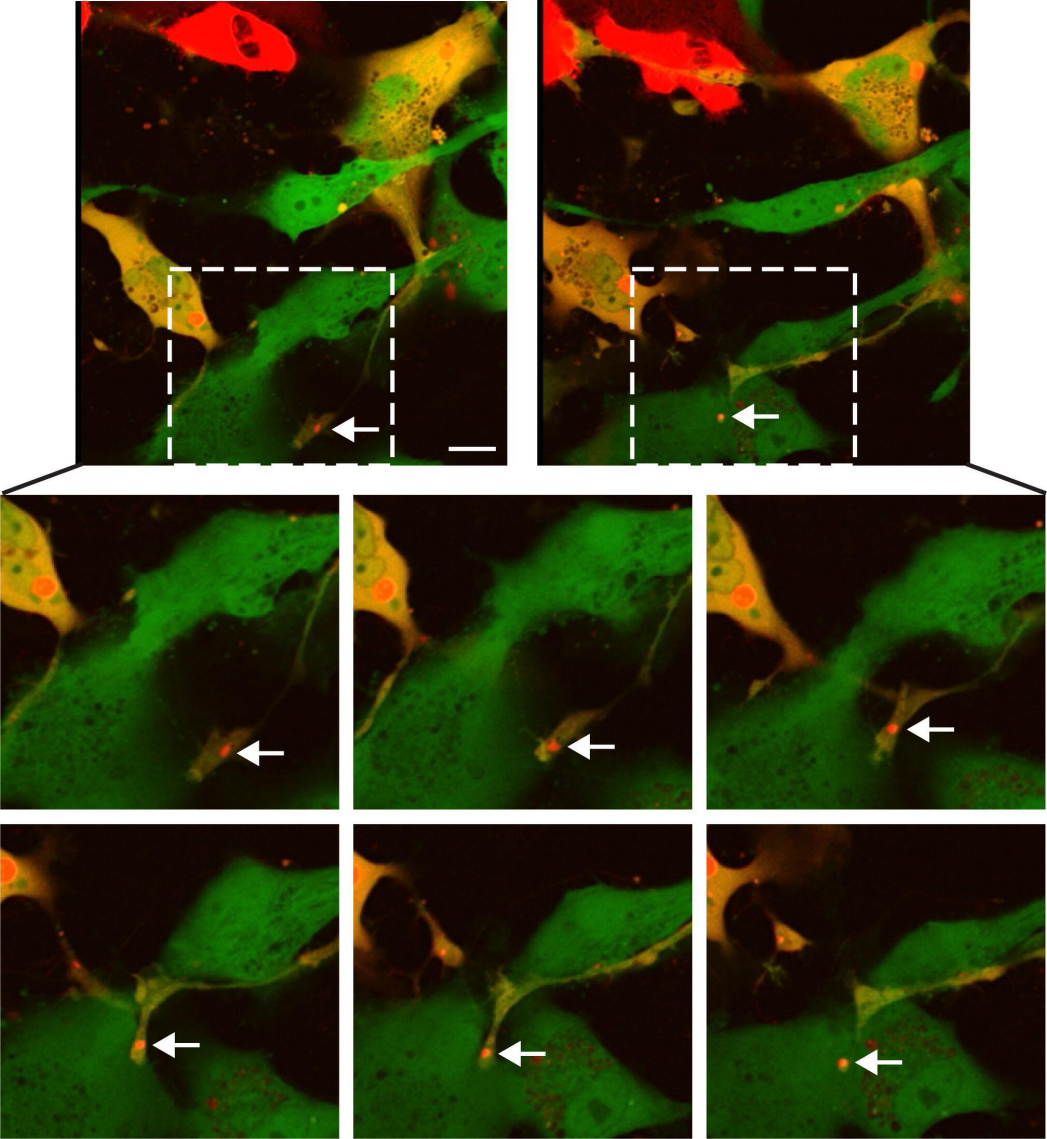
Inclusion bodies move from cell-to-cell across intercellular extensions in BEAS-2B cells. Live cell imaging was performed on BEAS-2B cells infected with rgHMPV and transfected with mCherry-HMPV *P* protein-expressing plasmid. Live cell imaging was performed 48 hpi and images were captured every 1 min (representative images are shown). White arrows show an inclusion body (red) at the end of an intercellular extension moving directly from cell-to-cell. Scale bar = 10 µm.

**Fig 7:**
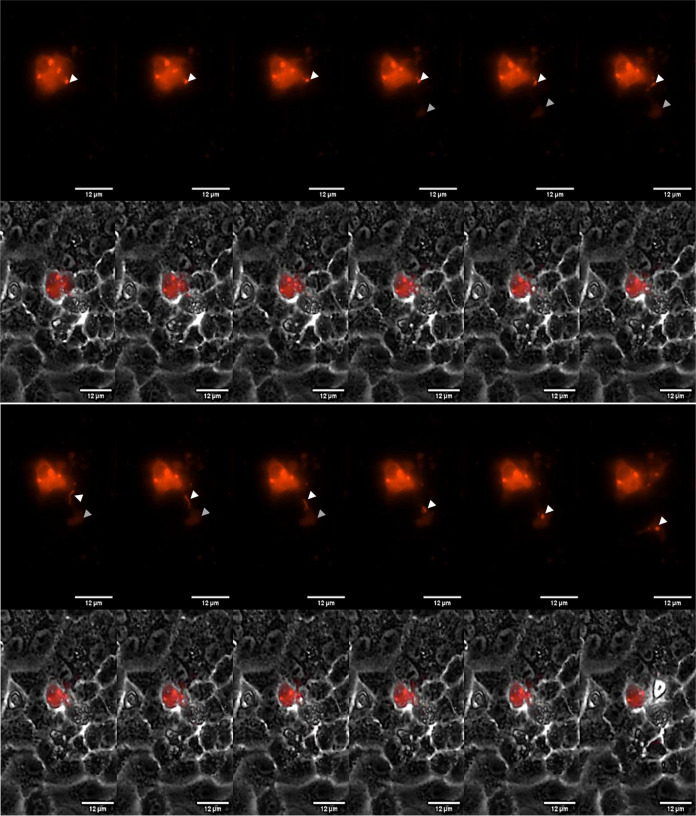
Transfer of an inclusion body from an HMPV-infected Vero E6 cell to a nearby noninfected cell. Representative images of the transfer of an IB from a cell infected with HMPV mCherry-P to a nearby uninfected cell (MOI = 1 and 72 hpi) obtained by live cell imaging. In the upper panels, the red signal corresponds to mCherry-P that was observed both in the cytoplasm and incorporated into IBs. In the bottom panels, phase contrast images merged with the red signal of mCherry-P are shown. The white arrowheads show a peripheral IB with a radius of 1.2 µm, which moves toward the right side of the cell with a mean velocity of 0.357 µm/s, median velocity of 0.273 µm/s, and a standard velocity of 0.385 µm/s. Gray arrowheads indicate regions with initial transfer of red dye, suggesting opening of a pore. Once the IB reached the right side of the cell, it was incorporated into a previously formed intercellular extension that connects the infected cell with an uninfected cell that was not immediately adjacent. Subsequent transfer of the whole inclusion body occurred at a maximum speed of 1.967 µm/s with a concomitant change in shape and length, reaching a maximal of 3.397 µm long. Once the inclusion body was fully transferred to the cytoplasm of the newly infected cell, it recovered the initial radius of 1.3 µm. The total distance of the inclusion body marked with the white arrowheads reached 12.682 µm. Images were captured every 1 min for a total of 24 h using a Lionheart FX automated microscope, with the representative images shown all taken over a 1-h period. All movement and size parameters were obtained using the FIJI plugin TrackMate.

HMPV can spread directly from cell-to-cell in the presence of neutralizing antibodies and independently of attachment factors that are needed for virus particle entry into cells, which argues that the F protein on the surface of virus particles is not required for this mode of spread. Intercellular extensions are important for mediating direct cell-to-cell spread of HMPV and both +RNA and −RNA have been detected in extensions suggesting that these structures play a role in the direct intercellular transfer of virus components [(3, 4), Fig. 4]. HMPV genomic RNA is tightly encapsidated with the N protein to which P, L, and M2-1 proteins are bound, providing all the needed components for vRNA synthesis. We hypothesized that HMPV genomes with the accompanying replication proteins are directly transported from cell-to-cell through intercellular extensions by movement of IBs. To test this hypothesis, we performed live cell imaging of HMPV-infected cells to track labeled mCherry-P protein using two different approaches. First, BEAS-2B cells infected with EGFP-expressing rgHMPV were transfected with mCherry-P protein and subject to live imaging. An example of this imaging (Fig. 6) shows an IB at the tip of an intercellular extension move directly from an infected cell-expressing mCherry-P to a distant infected cell expressing only GFP. The movement of an IB occurred in this case from an infected cell to another infected cell. Intercellular extensions induced in BEAS-2B cells are longer than those that form in Vero cells (4), so we also analyzed IB movement in a confluent layer of Vero cells. We infected Vero cells with a recombinant HMPV virus expressing mCherry-P and 72 hpi live cell imaging was performed. One example is shown in Fig. 7, where an IB from an infected cell moves along an intercellular extension connecting to a distant uninfected cell. As the IB moves into the intercellular extension, the shape changes from round-like with radius of 1.2 µm to a more snake-like structure reaching a length of 3.397 µm, thus supporting the fluidity and liquid-like nature of these structures as recently described (18). In addition, the velocity of the IB increased from 0.357 µm/s when it was located inside the infected cell to 1.967 µm/s as it passed across the extension. While the mechanisms and host factors involved in the movement of HMPV IBs remain to be explored, the data suggest that the mechanisms of transport of IBs through intercellular extensions and inside the cell are different. Upon transfer to the new cell, the IB re-attained its round-like shape. These results indicate that intercellular extensions formed upon HMPV infection in two different cell lines, BEAS-2B and Vero cells, can reach infected and noninfected cells and mediate direct transfer of IBs, which are known to contain vRNA and RNPs.

## DISCUSSION

Viruses have evolved several mechanisms that allow spread of infection directly from cell-to-cell independent of virus particle release. HMPV spread was previously shown to occur, partially, by a mechanism of direct cell-to-cell spread (4, 31), though the nature of the viral components involved in the spread was unclear. Intercellular extensions and a branching network of filaments were seen as a hallmark of HMPV infection in human bronchial airway epithelial cells. These structures are actin based and disruption of actin dynamics inhibited the formation of both the extensions and filaments, significantly decreasing direct cell-to-cell spread of HMPV (4). Here we used imaging techniques to better understand how intercellular extensions and branching filaments contribute to HMPV spread. Our data suggest that HMPV buds in a filamentous form that is rich in F protein and contain only the viral genome, but not the antigenome. Filamentous rod-like particles have also been reported by EM of HMPV isolates (34). These filaments emerge from the cell body and from intercellular extensions, and touch neighboring cells, potentially facilitating infection of the contacted cell. In addition, we show that intercellular extensions in HMPV-infected cells allow transfer of cytoplasmic content and IBs directly between cells. As IBs contain both viral genomic material and the needed components to facilitate viral transcription and replication, this would also be a mechanism to spread viral infection.

Intercellular extensions, defined mostly as filopodial bridges or tunneling nanotubes (TNTs), have been widely reported by fluorescence microscopy for several viruses; however, a limited number of reports studied these structures at the ultrastructural level. Our EM analysis revealed changes in the morphological characteristics of extensions following HMPV infection (Fig. 1 and 2). Intercellular extensions were detected in both mock-infected and HMPV-infected BEAS-2B cells as previously reported (4), but while the surface of extensions in mock-infected cells is smooth, extensions in HMPV-infected cells are covered with filamentous structures that are arranged in a parallel fashion and extend along the length of the extension (Fig. 1B and 2A). EM imaging of intercellular extensions induced by Sindbis virus (SINV) in Vero cells showed virus-sized particles on the surface but not parallel filaments (33). Parallel fiber bundles in TNTs have been described in uninfected A549 cells by transmission EM (TEM) but no further examination was done in influenza A virus (IAV)-infected cells (29). Bundles similar to those seen in infected BEAS-2B cells have been reported in neuronal cells as actin filaments (35). It is thus possible that these parallel filament bundles are filamentous actin-based structures induced following HMPV infection. Most recently, two studies using high-resolution EM of SARS-CoV-2-infected cells revealed intercellular filopodial bridges with virus-like particles adhering on their surface and budding particles at the tip of the filopodia (31, 36). Our study shows that vesicles of different sizes were also seen on the surface of extensions in HMPV-infected BEAS-2B cells and residing on top of the bundles (Fig. 1 and 2); however, the nature of these vesicles and whether they play a role in the transport of virus components across extensions remains to be determined. Another structure that was prominent in HMPV-infected cells is the shorter filaments that emanate from the intercellular extensions and the cell body. These filaments have an average length of 2 µm but can exceed 10 µm in some cells (Fig. 1C). Our results show that as infection progresses, HMPV F protein becomes highly concentrated in these filaments, which are rich in vRNA (Fig. 2through 4). Interestingly, the positive-sense RNA antigenome was not concentrated in the budding filaments, strongly suggesting that these structures represent mature filamentous virus particles being shuttled on extensions. Several viruses that are pathogenic to humans bud in a filamentous form, including influenza virus, RSV, and parainfluenza virus type 2 (PIV2) (37 – 39). Interestingly, multiple filamentous particles were seen touching a target cell (Fig. 3). A cluster of filamentous particles at the surface of filopodia that can reach 10 µm in length have been shown for the closely related RSV (28, 40). This indicates evolutionary similarities of this form of virion budding for pneumoviruses. Interestingly, a recent study using cryo-electron tomography showed that the interior of filamentous RSV virions is heavily packed with nucleocapsids suggesting polyploid virions (41). Given the size of the budding HMPV filaments, it is thus possible that these long filamentous particles can also contain multiple nucleocapsids.

Our results indicate that intercellular extensions in infected cells open to allow cytoplasmic communication between cells as seen by the transfer of the Alexa Fluor dye 594 (Fig. 5). However, dye transfer occurred at a very low rate, suggesting that intercellular extensions in BEAS-2B cells are not open ended and that HMPV infection triggers opening of the extensions. For alphaviruses, intercellular extensions involved in direct cell-to-cell spread of virus particles did not allow the transfer of a plasma membrane protein or a soluble cytosolic marker in infected Vero cells (33). Interestingly, it has been shown that TNTs in uninfected lung epithelial cells (A549) allow transfer of membrane components and mitochondria between cells (29), suggesting differences in the properties of these intercellular structures between epithelial cells of different origin. In addition, it is possible that there is selectivity in the cargo being transferred across intercellular extensions in epithelial cells. One of the earlier reports of TNTs in neuronal cells showed that while membrane and cytosolic proteins are transferred across TNTs, passive passage of soluble cytoplasmic molecules such as GFP did not occur (42). Regardless of the open- or close-ended nature of intercellular extensions in different cellular systems, they provide a unique platform for viruses to spread from cell-to-cell directly. This has been shown for a number of virus systems, although the mechanisms of how viruses use such extensions for direct spread of infection vary. Direct cell-to-cell spread of measles virus has been shown to occur across intercellular processes in neuroblastoma cells (43). Transfer of whole virus particles, such as HIV-1, murine leukemia virus (MLV), and herpesviruses can occur across extension-like structures (32, 44, 45). Direct passage of viral components have also been shown to occur across intercellular extensions including the transfer of viral proteins and genomes along TNTs in cells infected with influenza virus and porcine reproductive and respiratory syndrome virus (PRRSV) (29, 46). Usage of TNTs by SARS-CoV2 has also been recently reported and it has been suggested that virus particles can move on the surface of these tubes, although virus-like vesicles were detected inside the TNTs, suggesting that transfer of particles may occur via more than one route (34).

Live imaging analysis of infected BEAS-2B and Vero cells showed movement of mCherry-P tagged IBs from infected cells to neighboring cells across intercellular extensions (Fig. 6 and 7), with the fluid motion and changes in shape observed consistent with the characterization of HMPV IBs as phase separated regions (18). To our knowledge, this is the first report showing direct passage of IBs across extension-like structures between cells. Interestingly, we observed that IB and dye transfer did not always occur from one cell to the closest neighboring cell, raising the question of what cellular signals may affect the targeting of an intercellular extension. Transfer of IBs across intercellular extensions would offer a direct and protected route of viral spread and allow several advantages for infection (1). Infection by direct genome spread is faster than cell-free infection and more efficient since direct insertion of multiple genomes along with their replication machinery occurs, overcoming the rate-limiting fluid diffusion phase with release, transmission, and entry. In addition, infection becomes more efficient with the probability of successful infection highly increasing when viral genomes are directly inserted virus-to-cell, avoiding virus-cell interactions that may limit productive infection. Direct spread offers evasion of physical and immunological barriers including proteases, phagocytic cells, neutralizing antibodies in addition to pharmaceutical targeting [reviewed in reference (1)]. Spread of HMPV infection in the presence of neutralizing antibodies occurs in both BEAS-2B cells and three-dimensional human airway tissues, and also in the absence of virus attachment factors, strongly suggesting that transfer occurs independent of HMPV F protein on the virion surface (4, 47). Thus, direct movement of IBs across extensions would offer an intriguing novel mechanism of pneumovirus spread within the host. One limitation of our live imaging experiments is that, due to photodamage, the cells did not survive long enough to detect productive infection in the target cells following IB transfer and to follow the fate of the IB in the target cells. Further examination is needed to confirm that IBs moving along extensions are active replication bodies that are able to continue the viral replication cycle to spread HMPV infection. However, our previous demonstrations of direct cell-to-cell spread resulting in productive infection are consistent with the model that IB transfer leads to productive infection of the target cell (4).

A recent report showed that HMPV IBs are dynamic structures with liquid-like properties that can undergo fusion and fission events (18). The fluidity of these structures during cell-to-cell transfer was clear as an IB underwent significant changes in size, shape, and speed as it passed across an intercellular extension from an infected Vero cell to an uninfected cell (Fig. 6). The size of HMPV IBs increases significantly from 24 to 72 hpi, indicating that cellular and viral components most likely change and build up in these structures as infection proceeds (18). Interestingly, fission levels increase significantly between 24 and 72 hpi with smaller bodies seen coming off the main IB. It remains to be determined if there is selectivity in the IB subpopulations being transferred across intercellular extensions. In addition, the host factors involved in trafficking of HMPV IBs are still unknown. Recently, Rab11a was found to play a role in the transfer of IAV vRNPs from cell-to-cell independent of full particle assembly (32); however, Rab11a does not play a role in HMPV IB trafficking (8). The actin cytoskeleton was shown to be essential for coalescence of smaller IBs to larger IBs, so HMPV IBs could utilize one of the actin motor proteins or transport vesicles associated with the actin cytoskeleton to be transported across the actin-based extensions.

Our findings challenge the concept of what an infectious unit is. HMPV remains primarily cell-associated and is challenging to retrieve high virus titers *in vitro*. The data presented here strongly suggest that HMPV buds mainly as cell-associated filamentous particles at cell-cell contact sites. Multiple budding filaments were seen contacting neighboring cells supporting the notion that infection by multiple viral genomes at the same time, referred to as *en bloc* transmission, can occur through these structures (22). This mode of spread offers several advantages to the virus to ensure rapid and more efficient infection at sites of cell-cell contact (22, 48 – 51). In addition, spread by this manner may not have the same requirements for cell-free infection in terms of attachment factors needed or sites on the F protein being targeted by neutralizing antibodies; however, this hypothesis requires further investigation. Our live imaging analysis also revealed direct transfer of HMPV genomes present in IBs across intercellular extensions, raising the idea that direct spread of infection between host cells by transport of genome-containing replication bodies can occur. These findings suggest that infected cells are critical for efficient spread of viral infection inside a host and thus influences the antiviral strategies that can be used to target HMPV infection.

## Data Availability

Microscopy images and quantitation data are available at Mendeley Data, V2, doi: 10.17632/swx3cvfzz5.2.
